# Coexistence of Benign Brenner Tumor with Mucinous Cystadenoma in an Ovarian Mass

**DOI:** 10.30699/ijp.2020.104017.2052

**Published:** 2020-07-16

**Authors:** Farzaneh Nazari, Zahra Dehghani

**Affiliations:** 1 *Department of Gynecology and Obstetrics, School of medicine, Bushehr university of medical sciences, Bushehr, Iran*; 2 *Department of Pathology, School of Medicine, Bushehr University of Medical Sciences, Bushehr, Iran*

**Keywords:** Brenner tumor, Mucinous tumor, Ovarian cancer

## Abstract

Ovarian cancer is the most lethal gynecologic malignancy. The surface epithelial tumor is the most common type of ovarian cancer. Among these, the mucinous tumors account for 10-15% of ovarian tumors. Mucinous ovarian tumors are among the most difficult ovarian neoplasms for surgical pathologists to interpret. Mucinous tumors sometimes coexist with other surface epithelial tumors. Therefore, making the accurate diagnosis of the mucinous tumors is essential. On the other hand, association of Brenner tumors with other neoplasms is rare. Ovarian Brenner tumor has always been discussed by pathologists as an enigmatic tumor, because of its rarity and disputed histogenesis. Here, we reported a case of large mucinous cystadenoma with Brenner component.

## Introduction

Ovarian tumors are common forms of neoplasia in women. Ovarian cancer is the seventh most commonly diagnosed cancer among women in the world (about 30% of female genital cancers). Ovarian cancers have the highest cancer-related mortality among gynecological cancers, and those are usually asymptomatic in the early stage. Moreover, the symptoms are vague and unspecific (1,2). Ovarian tumors are classified according to its origin into three main groups of epithelial, stromal and germ cell tumors. Epithelial tumors are the most common type (3). These tumors account for about two-thirds of all primary ovarian tumors. Transitional cell tumors of the ovary (an uncommon subtype of the surface epithelial tumor) are rare neoplasms and account for about 2% of all ovarian tumors (4). About 20% of these tumors occur together with mucinous or serous cystadenomas or benign teratomas (5).

We are reporting a case of coexistence of mucinous cystadenoma with Brenner tumors to make the pathologists and gynecologists aware about the occurrence of such combined ovarian tumors.

##  Case Report

A 58-year-old, nulliparous, post-menopausal woman was admitted to a hospital with a chief complaint of abdominal pain and distension for 6 months. Abdominal pain had started six months ago with a gradual onset, progressive course, diffuse, and not associated with any gastrointestinal symptoms. She had primary infertility (only one miscarriage 30 years ago). She was post-menopausal for 15 years. Apart from being a heavy water-pipe smoker, her medical history was uneventful. Vital signs were stable. A huge abdominopelvic mass was palpated in physical examination. The borders were irregular and not tender in palpation. Intestinal sounds were audible. The vaginal examination was unremarkable. Ultrasound examination showed a solid cystic mass (104×115 mm) with multiple thick septa and internal echo in mid pelvis suggestive of an ovarian complex cyst. MRI images revealed a multiloculated cystic mass (104 ×200 × 265) in the pelvis with extension to the abdominal cavity, suggestive of ovarian mucinous cystadenoma or carcinoma. Tumor markers (CA125, AFP, HE4, LDH, CA19-9, CEA) were in normal limits. . Laboratory data and Chest X‑ray had normal findings. A written informed consent was obtained from patient. After general anesthesia, abdominopelvic exploration was done with a midline incision. There was a huge mass arising from the right ovary with a high possibility of mucinous cystadenoma. The mass was ruptured when removing (Figures 1 and 2). Total hysterectomy, bilateral salpingo‑oophorectomy and peritoneal washing was performed due to a high possibility of malignancy.

**Fig. 1a:**
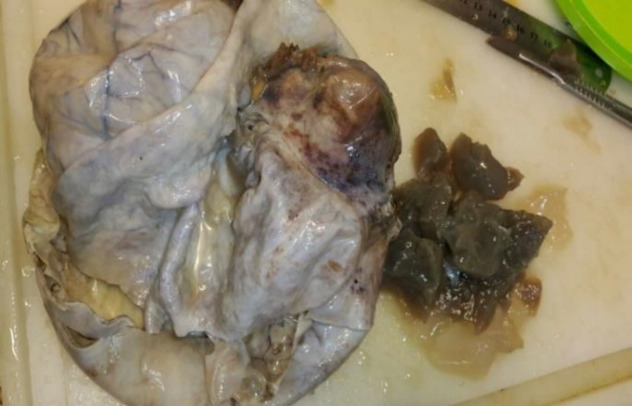
Gross specimen

**Fig. 1b:**
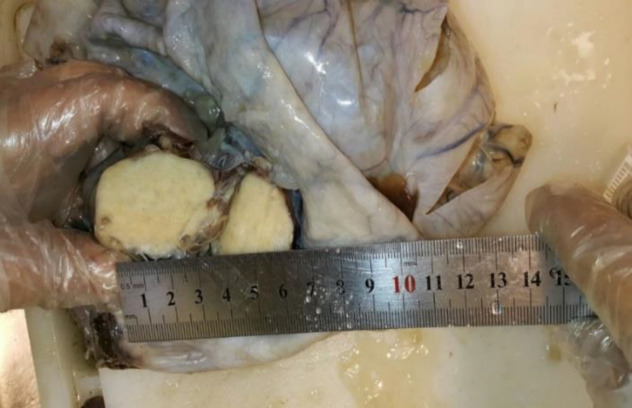
A large multilocular cyst filled with mucinous material and a well-defined firm creamy mass in cystic cavity

**Fig. 2a:**
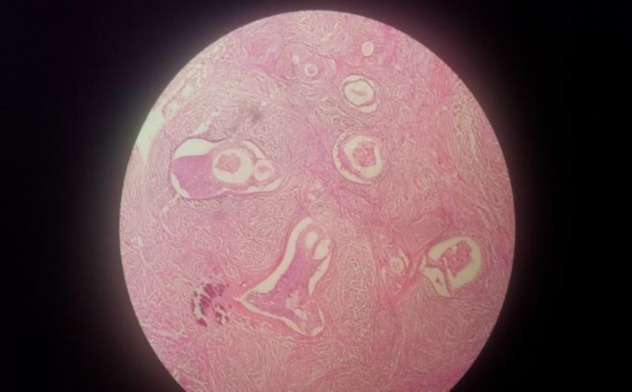
Well‑defined nests of transitional epithelium within a fibrous stroma (low power)

**Fig. 2b:**
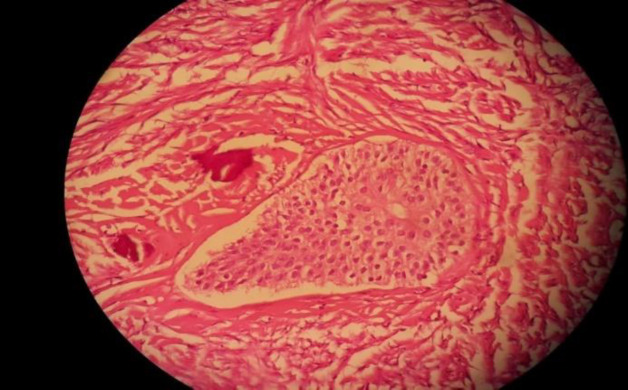
A well‑defined nest of transitional epithelium within a fibrous stroma (high power)

**Fig. 2c:**
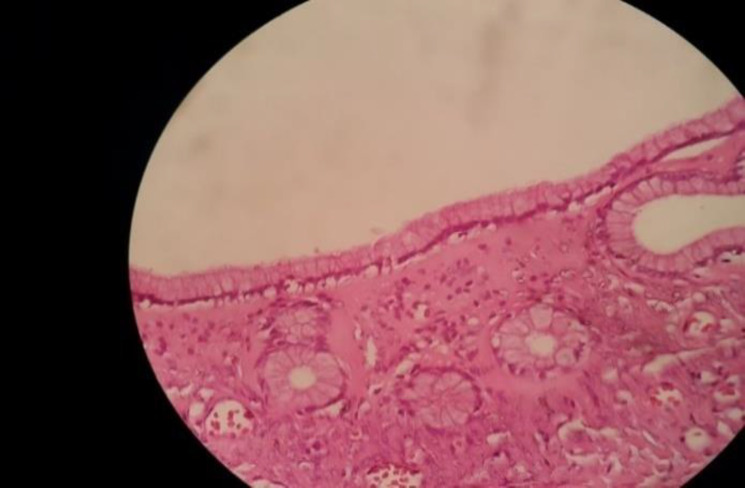
No cellular atypia was observed

Grossly, a 8×5×4 cm hysterectomy specimen with bilateral adnexa was observed. Furthermore, there was a ruptured right ovarian cyst measuring 20×13 cm with smooth external surface along with 5 cm long tube (Figure 1a). Also the size of left ovary was 3×2×1 cm along with 4 cm tube. Cut section of uterus, cervix, left ovary and tube appeared normal. Figure1b shows the cut section of the right ovarian cyst. There was a large multilocular cyst filled with mucinous material and a well-defined firm creamy mass. Figure 2 demonstrates well‑defined nests of transitional epithelium within a fibrous stroma. These histological features are in accordance with mucinous cystadenoma with Brenner component. Furthermore, she was followed up for 2 years without evidence of tumor recurrence and metastasis by physical examination and abdominopelvic ultrasound scan.

## Discussion

Although the relationship between ovarian mucinous tumor and Brenner tumor is well known, the coexistence of two types of ovarian tumors has rarely been reported (5). Brenner tumors are formerly known as transitional cell tumors because of their histologic similarity to the urothelium. The average age at presentation is 50 years, with 71% of the patients older than 40 years (4). Sometimes these tumors are found incidentally on pathologic examination for oophorectomy, which is performed for other reasons. Usually, Brenner tumors do not have any symptoms. Sometimes there was abdominal pain or vaginal bleeding (6). Histologically, most Brenner tumors are benign; malignant tumors are very rare, around 2-5% of cases (7). Brenner tumor is rare with changing histological criteria. There is no confirmed tumor marker for malignant Brenner tumors. Even though ovarian Brenner tumors do not have hormonal secretions, there are descriptions of steroid hormone-producing Brenner tumors (8). The histogenesis of Brenner tumors has always been interesting despite a great controversy (9). Some of the theories suggest an origin from the granulosa cells of the Graafian follicle or follicular epithelium, rete ovarii and mesonephric remnants, Walthard cell rest, and coelomic epithelium (10). The most widely believed histogenesis of these tumors supports their derivation from ovarian surface epithelium or pelvic mesothelium. Accompaniment of ovarian Brenner tumors and other tumors arising from surface epithelium (mucinous and serous cystadenomas) strongly favors a surface epithelial histogenesis (11,12).

 Microscopically, Brenner tumors are character-ristically made of abundant dense fibroblastic stroma with solid and cystic epithelial nests of transitional cells resembling those of urinary bladder (10,13). The cells show oval nuclei with distinct nucleoli and frequent nuclear grooves giving a coffee-bean appearance. The microcysts are lined by metaplastic columnar epithelium with eosinophilic secretions in the lumen. This criterion suggests mucinous cystadenoma. The stroma may show focal hyalinization and calcific deposits. The presence of both epithelial and stromal components has been described as the features of Brenner tumors. Polygonal cells in clusters or single cells with moderately pleomorphic nuclei and multinucleate cells with variable mitotic figures revealed cytologic features of borderline or malignant Brenner tumors (11,14,15).

The general management of Brenner tumors in postmenopausal women is abdominal hysterectomy with bilateral salpingo- oophorectomy. This was performed for our patient because of her menopausal status and the possibility of malignancy.

## Conclusion

Ovarian masses, particularly those looking benign, should be thoroughly examined for presence of malignant components. Late diagnosis or misdiagnosis of accompanied malignancy may lead to devastating consequences.
